# In This Issue

**Published:** 2004

## SCREENING AND BRIEF INTERVENTION IN PRIMARY CARE SETTINGS

By routinely screening all patients for potential alcohol problems and intervening when needed, primary care practitioners can play an important role in reducing the rate of alcohol-related problems in the general population. As Dr. Michael F. Fleming explains, however, screening and brief intervention (i.e., one-time or repeated short counseling sessions designed to minimize alcohol use and related problems) continue to be underutilized in primary care settings. Dr. Fleming proposes that varied levels of screening and brief intervention can be implemented by primary care practitioners, depending on patient and physician factors, including patients’ co-occurring medical or psychiatric problems, physician skills and interest, and the amount of time available. Strategies that may help increase physicians’ use of screening and brief intervention include educational approaches such as skills-based role-playing, performance feedback, clinical protocols, clinic-based education, financial incentives, and training by credible experts. (pp. 57–62)

## SCREENING AND BRIEF INTERVENTION IN THE EMERGENCY DEPARTMENT

Almost one-third of patients treated in emergency departments (EDs) and about half of severely injured trauma patients have alcohol problems, according to Drs. Gail D’Onofrio and Linda C. Degutis. However, only a small proportion of ED patients with alcohol-related problems are diagnosed appropriately or receive the necessary treatment. Several studies have shown that brief interventions delivered in the ED or trauma unit can reduce subsequent drinking, drinking and driving, and other adverse consequences. The challenge remains, however, how to integrate effective brief interventions in the real-world ED setting, with its time and personnel constraints. Innovative approaches such as computer-assisted screening and intervention may help solve some of these problems. (pp. 63–72)

## LEGAL BARRIERS TO ALCOHOL SCREENING IN EMERGENCY DEPARTMENTS AND TRAUMA CENTERS

Research has shown that many patients being treated in emergency departments (EDs) and trauma centers have alcohol-related problems and could benefit from screening and brief intervention. Yet few ED and trauma center physicians routinely screen all patients for alcohol-related problems. According to Judge Linda Chezem, one reason for the lack of screening in this setting may be the barriers posed by insurance laws. Currently, insurance statutes based on the 40-year-old Uniform Accident and Sickness Policy Provision Law (UPPL) allow insurance carriers to deny benefits to a patient whose injuries or conditions occurred when he or she was intoxicated. Judge Chezem reviews the history of the UPPL provisions and discusses their implications for patients and health care providers, as well as efforts under way to repeal these provisions. (pp. 73–77)

## SCREENING AND BRIEF INTERVENTION IN PRENATAL CARE SETTINGS

Although research has proven that drinking alcohol during pregnancy can be harmful to the fetus, some pregnant women continue to drink. No universally safe level of prenatal alcohol use has been determined. Thus, it is important for clinicians to be able to identify which women are drinking or might be likely to drink during pregnancy to help them modify their alcohol use. Dr. Grace Chang describes a screening instrument shown to be effective for identifying a range of alcohol use with this population: the T-ACE, a four-item questionnaire based on the CAGE assessment tool. Once pregnant women are warned that they are consuming alcohol at potentially problematic levels, they tend to be receptive to changing their drinking behavior, making them ideal candidates to receive some type of brief intervention. The author reviews studies that examined the effectiveness of brief interventions with pregnant women in prenatal care settings. In general, these studies found that pregnant women receiving brief interventions significantly reduced their alcohol consumption. (pp. 80–84)

## SCREENING AND BRIEF INTERVENTION IN THE CRIMINAL JUSTICE SYSTEM

About 40 percent of offenders on probation, in State prisons, or in local jails report that they had been using alcohol at the time of their offense, and each year about 1.4 million Americans are arrested for driving while intoxicated (DWI). Thus, the criminal justice population is an important target for screening and brief interventions for alcohol problems. Dr. Sandra Lapham reports, however, that although screening is mandatory for DWI offenders in most States, no nationwide standards exist for the screening of non-DWI offenders. Moreover, current screening procedures have limitations that may reduce their effectiveness among criminal justice populations. Factors that may impair the effectiveness of screening and brief interventions in the criminal justice system are the potentially coercive nature of screening in this setting, financial constraints on the criminal justice system, and the presence of coexisting mental illnesses in offenders. (pp. 85–93)

## BRIEF INTERVENTION IN COLLEGE SETTINGS

College students have high rates of alcohol use and misuse and suffer the negative consequences of this behavior, such as physical illness, academic problems, and aggressive confrontations with others. Several studies have shown that brief interventions with high-risk college students are successful in reducing alcohol consumption and/or the related consequences, as reviewed by Dr. Mary E. Larimer, Ms. Jessica M. Cronce, Dr. Christine M. Lee, and Dr. Jason R. Kilmer. The authors also examine the effectiveness of screening tools—such as the CAGE questionnaire, the Michigan Alcoholism Screening Test, the Young Adult Alcohol Problems Screening Test, the College Alcohol Problems Scale–revised, the Rutgers Alcohol Problem Index, NIAAA’s alcohol consumption question sets, and the Alcohol Use Disorders Identification Test—in detecting problematic alcohol use and associated disorders among college students. College campuses offer several opportunities to implement screening and intervention, such as through health care facilities and local emergency rooms, as well as via campus judicial or grievance systems that mandate assessment and interventions for students who violate school alcohol policies. (pp. 94–104)

